# Steam catalytic cracking and lump kinetics of naphtha to light olefins over nanocrystalline ZSM-5 zeolite

**DOI:** 10.1039/d3ra03157h

**Published:** 2023-08-31

**Authors:** Emad N. Al-Shafei, Ali N. Aljishi, Zaidoon M. Shakoor, Mohammed Z. Albahar, Mohammad F. Aljishi, Ahmed Alasseel

**Affiliations:** a Research and Development Center, Saudi Aramco Dhahran 31311 Saudi Arabia EMAD.SHAFEI@ARAMCO.COM emadnaji@gmail.com; b Chemical Engineering Department, University of Technology Baghdad Iraq

## Abstract

This study investigates the reaction pathways and kinetics to comprehend the catalytic cracking of dodecane, a heavy naphtha model compound, over the nanocrystalline ZSM-5 catalyst in the presence and absence of steam with the aim of increasing olefin production. The nanocrystalline zeolite was characterized using XRD and BET, and the surface acidity was measured by NH_3_-TPD and Py-FTIR. The steam treated ZSM-5 contributed to an increase in pore volume with extra-framework alumina, resulting in highly catalytic active sites and hence higher olefin selectivity. The high conversion of dodecane (>90%) was achieved during catalytic cracking in the presence and absence of steam. In the presence of steam, the short pores of nano ZSM-5 led to an increase in the naphtha-to-olefin conversion with lesser dry gas and coke formation. The activation energies of primary cracking in the presence and absence of steam were slightly different. Lower activation energies through secondary cracking routes and higher reaction rate constants were obtained *via* assisted-steam catalytic cracking, promoted the selectivity towards light olefin products. Meanwhile the hydrogenation and alkylation reactions toward LPG and C5+ were favored in the absence of steam. Moreover, the ZSM-5 nano zeolite pores promoted more β-scission reactions, resulting in higher selectivity towards ethylene and dry gas.

## Abstract 2

This study investigates the reaction pathways and kinetics to comprehend the catalytic cracking of dodecane, a heavy naphtha model compound, to light olefin, over the nanocrystalline ZSM-5 catalyst in the presence and absence of steam.

## Graphical abstract

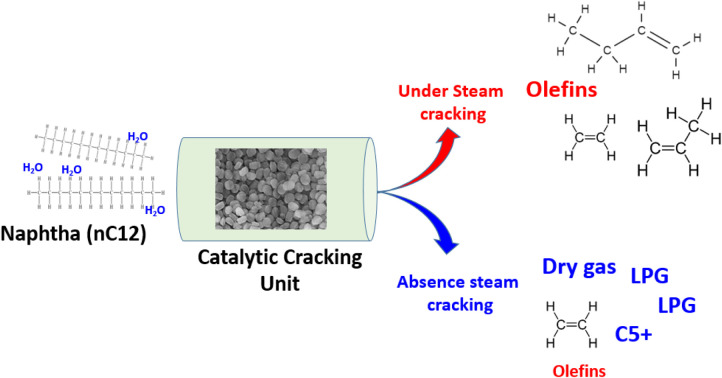

## 1. Introduction

The utilization and conversion of naphtha to high-value chemicals through catalytic cracking routes have received considerable attention in order to meet the high demand for basic valuable chemicals.^1–4^ Several studies have explored the non-assisted and steam-assisted catalytic cracking routes of full-range petroleum naphtha to increase light olefin and BTX (benzene, toluene, and xylene) production.^5–7^ Naphtha catalytic cracking has several advantages over conventional noncatalytic steam cracking.^8^ It operates at lower reaction temperatures, and the utilized catalyst contributes to obtaining higher selectivity towards desired products.^5,9^ Although a number of zeolite-based catalysts have been broadly studied for cracking naphtha to olefins, steam catalytic cracking has remained a challenging catalytic process. The presence of steam in the naphtha catalytic cracking reaction can impose an irreversible dealumination to the zeolite framework, leading to low olefin yield.^10,11^ Furthermore, it was found that ZSM-5 with micro-sized crystals is prone to more coke deposition in the pores.^12–15^ To overcome these challenges, zeolites are usually modified with rare-earth metals or phosphorus to decrease framework dealumination.^16–19^ Moreover, these modification techniques have been effective in increasing catalyst reactivity in the steam catalytic cracking of hexane to olefins. Further, the cyclization reaction route is less favored at 650 °C, resulting in more olefins.^20,21^ Furthermore, several studies have shown that the use of steam reduces coke formation over the catalyst during high-temperature hydrocarbon conversion.^8,11^ Yamaguchi *et al.*^20,21^ studied hexane steam catalytic cracking at 650 °C over ZSM-5 modified with phosphorus, and they achieved high olefin yields and low aromatics, suggesting that secondary reactions were suppressed in the presence of steam during conversion. Accordingly, it is understood that introducing steam during the catalytic cracking reaction promotes more primary cracking pathways, which lead to more olefin molecules leaving the pores and reduced cyclization reactions that form coke.^11^

Recently, more attention has been directed towards nanoscale zeolite as it has shown significant improvements in different hydrocarbon catalytic cracking reactions compared with microscale zeolite.^8,22^ Several studies have addressed the effect of nanoscale zeolites and showed higher activity with lower coke deposition,^15^ resulting in lower deactivation rates, which means longer time on the stream with high conversion.^17,22,23^ Furthermore, nanoscale zeolites have shorter path lengths inside the pores compared with normal-sized zeolite crystals, and this leads to faster diffusion rates, allowing the product to diffuse faster without undergoing further secondary reactions.^23–25^ Among these zeolites, nanoscale ZSM-5 showed the highest activity and pore-shape selectivity toward converting different hydrocarbons to olefins. Corma *et al.*^11^ studied the steam catalytic cracking of wide-range of paraffin (C_5_–C_12_) over ZSM-5 zeolite with crystal sizes ranging from 100 to 1000 nm. It was observed that nanocrystals of ZSM-5 resulted in lower coke deposition^11,15^ and lower yield of methane gas.^11^

In this research, dodecane catalytic cracking was carried out over nanoscale ZSM-5 zeolite in the presence and absence of steam. The objective of the study was to increase olefin yield and assess the effect of steam on the reaction pathways. Using long-chain dodecane rather than *n*C_6_ (ref. 26) and *n*C_7_ (ref. 35) as a model of heavy naphtha feedstock in catalytic cracking provided a range of intermediate products that are useful for studying the kinetics associated with pore shape selectivity performance. Various hydrocarbon monomolecular cracking reactions occurred over the catalyst surface and proceeded *via* protonation with increasing rates, especially due to faster diffusion enabled by the shorter cylindrical pore of nanocrystal zeolite.^27–29^ In this regard, the product components were grouped^30^ to build a seven-lump kinetic model to simulate the reactions controlled by diffusion inside nanocrystalline ZSM-5 zeolite, which has a shorter pore path length.

## 2. Experimental details

### 2.1 Zeolite preparation

Tetrapropylammonium hydroxide (TPAOH, 1 M), aluminum sulfate octahydrate, tetraethyl orthosilicate (TEOS), ammonium nitrate and reverse osmosis (RO) water were used to synthesise the MFI ZSM-5 zeolite catalyst. The molar ratio used for ZSM-5 preparation was 1 SiO_2_ : 0.02 Al_2_O_3_ : 58.57 H_2_O : 0.276 TPAOH. It was hydrothermally synthesized at 170 °C for 18 hours. Aluminium sulphate octahydrate (0.64 g) was added to 75.5 g of RO water and mixed for 1 h. Then, TPAOH (32.8 g) was added to the alumina solution and mixed for 1 h at room temperature. Thereafter, TEOS (10 g) was added to the mixture under stirring for 3 h; then, the solution was transferred to an autoclave cell for hydrothermal synthesis. The zeolite powder was collected using centrifugation and washed three times using RO water before drying at 90 °C in a vacuum oven for 18 h. Then, calcination was conducted at 550 °C for 5.5 h to remove the template, and the prepared ZSM-5 with a silica/alumina ratio of 50 was ion-exchanged with an ammonium nitrate solution (2.0 M) at 75 °C for 3 h. Finally, the zeolite was washed with RO water and dried before another calcination step at 550 °C for 5.5 h to obtain H-ZSM-5.

### 2.2 Zeolite characterization

The synthesized ZSM-5 zeolite was characterized based on nitrogen adsorption using ASAP 2420 from Micromeritics to study the textural properties. Moreover, X-ray diffraction (XRD) was applied using a Panalytical X'Pert PRO Diffractometer to determine the crystallinity. Furthermore, the effect of steam on the alumina framework was investigated by solid-state ^27^Al magic-angle spinning nuclear magnetic resonance (MAS NMR) using Varian 500 MHz. Moreover, the surface morphology was studied using the scanning electron microscopy (SEM) technique on an FEI Quanta 400 instrument. The zeolite acidity was determined by temperature-programmed desorption of ammonia (NH_3_-TPD) using a Micromeritics Autochem 2910 (ref. 31) and pyridine-FTIR using a Nicolet-6700 spectrometer.^32,33^

### 2.3 The catalytic cracking reactor apparatus

**Fig. 1:**
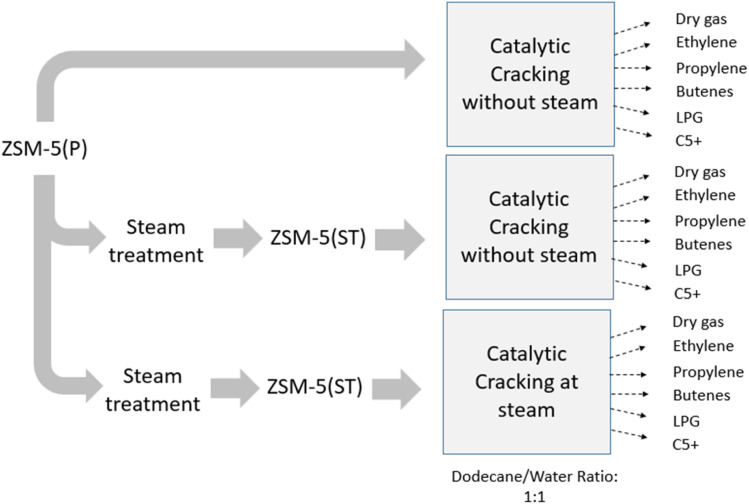
Scheme of ZSM-5(P) and ZSM-5(ST) experiments for dodecane catalytic cracking conversion.

Fig. 1 illustrates the investigation scheme of the ZSM-5(P) and steam-treated ZSM-5(ST) catalysts in the catalytic cracking of dodecane in the absence and presence of steam. The nanoscale ZSM-5(P) zeolite was treated with steam at 600 °C with RO water at a liquid hourly space velocity of 1.0 h^−1^ at STP using an HPLC pump for injection. The steam treatment of ZSM-5 was conducted for 6 hours with a nitrogen flow of 50 mL min^−1^; then, the HPLC pump was stopped, while the nitrogen flow was marinated for 2 hours prior and labelled ZSM-5(ST). The catalytic cracking reaction was carried out in a fixed-bed reactor under nitrogen flow at 10 mL min^−1^, while two HPLC pumps were utilized to inject water and dodecane at a liquid hourly space velocity (LHSV) of 1.0 h^−1^ each, resulting in a dodecane/water volume ratio of 1 : 1 for steam-assisted catalytic cracking over the ZSM-5(ST) catalyst. After each experiment, the zeolite was regenerated by flowing air at 100 mL min^−1^ and 650 °C for 2–3 h. On the other hand, in the absence of steam, only dodecane was fed to the reactor over the ZSM-5(P) and ZSM-5(ST) catalysts, separately. Catalytic cracking of dodecane was conducted at 500, 550, and 600 °C. In order to ensure full gas–liquid separation, a cold trap was used. A gas chromatograph (GC) equipped with a flame ionization detector (FID) and a thermal conductivity detector (TCD) was utilized to analyse the gas products, while the liquid hydrocarbon phase was separated from water and re-weighted. The hydrocarbon liquid phase was analyzed by gas chromatography-mass spectrometry (GC-MS). The product selectivities towards olefins (ethylene, propylene and butenes), dry gas (methane and ethane), LPG (propane, iso-butane, *n*-butane), C5+ (pentane and naphtha range of hydrocarbons) were calculated in weight percentage according to eqn (1) and (2) shown below.1
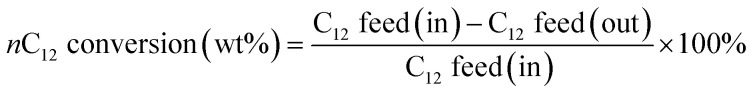
2
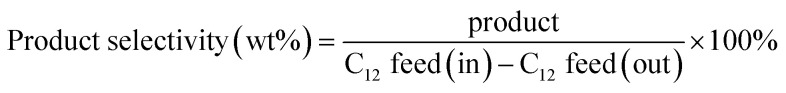


### 2.4 Kinetic modelling

**Fig. 2:**
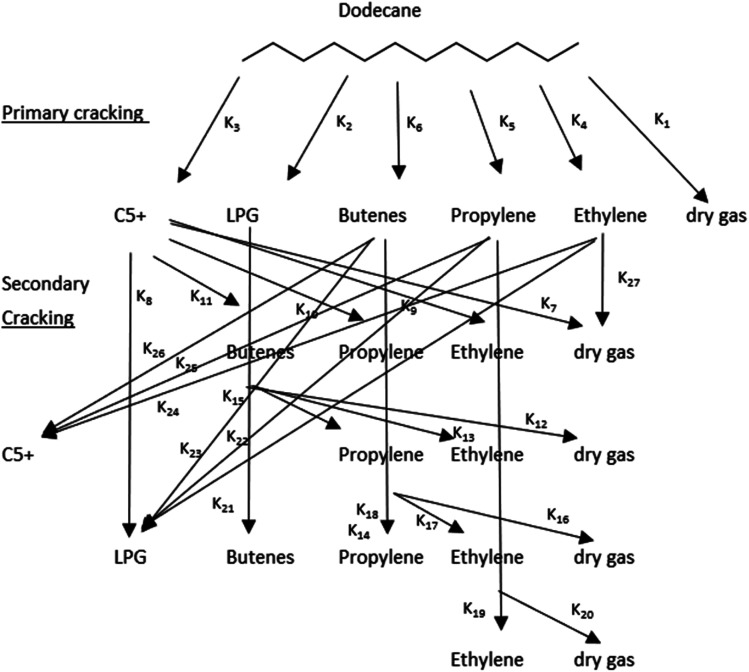
Lumping kinetics of the reaction pathway of *n*-dodecane cracking to olefins.

Lumping kinetic analysis was performed to study pore shape selectivity during dodecane catalytic cracking over nanoscale ZSM-5 in the absence and presence of steam. The lumping approach, in which product components are combined based on a number of well-defined groups, provides a flexible and robust means for predicting reaction pathways, product yields and conversion parameters. Multiple lumped models have been developed for the catalytic cracking of naphtha to olefins.^34,35^ In this study, a seven-lump kinetic model^36^ was used to evaluate the main product groups, including dry gas, LPG, C5+ and olefins (ethylene, propylene, butenes), resulting from the primary cracking of naphtha and secondary cracking of the intermediate byproducts *via* the zeolite pores. Dodecane catalytic cracking under selected conditions was modelled to proceed *via* the monomolecular and/or bimolecular reaction mechanism pathways, as illustrated in Fig. 2. For the seven-lump kinetic model, 27 rate constants were estimated by solving a system of differential equations. The differential equation (eqn (3)) employed to represent the difference in the molar flow rate (eqn (4)) of the reaction mixture within the fixed-bed reactor was as follows:3
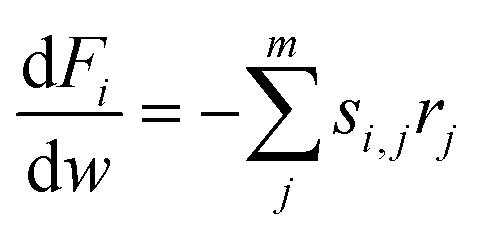

4 *r*_*j*_ = *K*_*i*_*I*

where *F*_*i*_ is the molar flow rate of the *i* component (mol h^−1^), *w* is the catalyst weight (g), *r*_*j*_ is the reaction rate per catalyst mass (mol h^−1^ per g cat), *C*_*i*_ is the molar concentration of the *i* component (mol m^−3^), and *s*_*i*,*j*_ is the stoichiometric coefficient for the *i* component participating in the *j* reaction. The Arrhenius equation (eqn (5)) was applied to represent the variation of the reaction rate constants with temperature and to estimate the activation energies and pre-exponential factors, as shown below:5
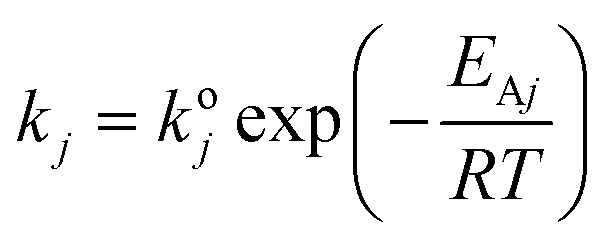
where *R* is the gas constant 8.314 (J mol^−1^ K^−1^), *T* is the temperature (K), *k*^o^_*j*_ is the pre-exponential factor (m^3^ g^−1^ per cat. h), and *E*_A_ is the activation energy (J mol^−1^). The model involved solving seven ordinary differential equations, one for each component. A differential evaluation–optimization algorithm was used to estimate a global optimum set of kinetic parameters by minimizing the objective function (minimum mean relative error), and the mean relative error (MRE) was calculated using eqn (6). All computations in this study were performed using programs coded in the Matlab 2015a software. The Genetic Algorithm optimization technique was used to estimate the optimum set of kinetic parameters, whilst the fourth-order Runge–Kutta integration method^37^ was used to integrate the simultaneous ordinary differential equations. Matlab functions *GA* and *ode45* differential equation solver were used for optimization and integration. The values of the pre-exponential factors and activation energies^38,39^ were evaluated to mimic the catalytic performance in the catalytic cracking of dodecane in the absence and presence of steam.6
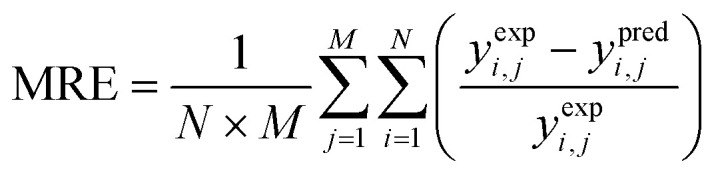


## 3. Results and discussion

### 3.1 Zeolite morphology, textural properties and the Al framework

**Fig. 3:**
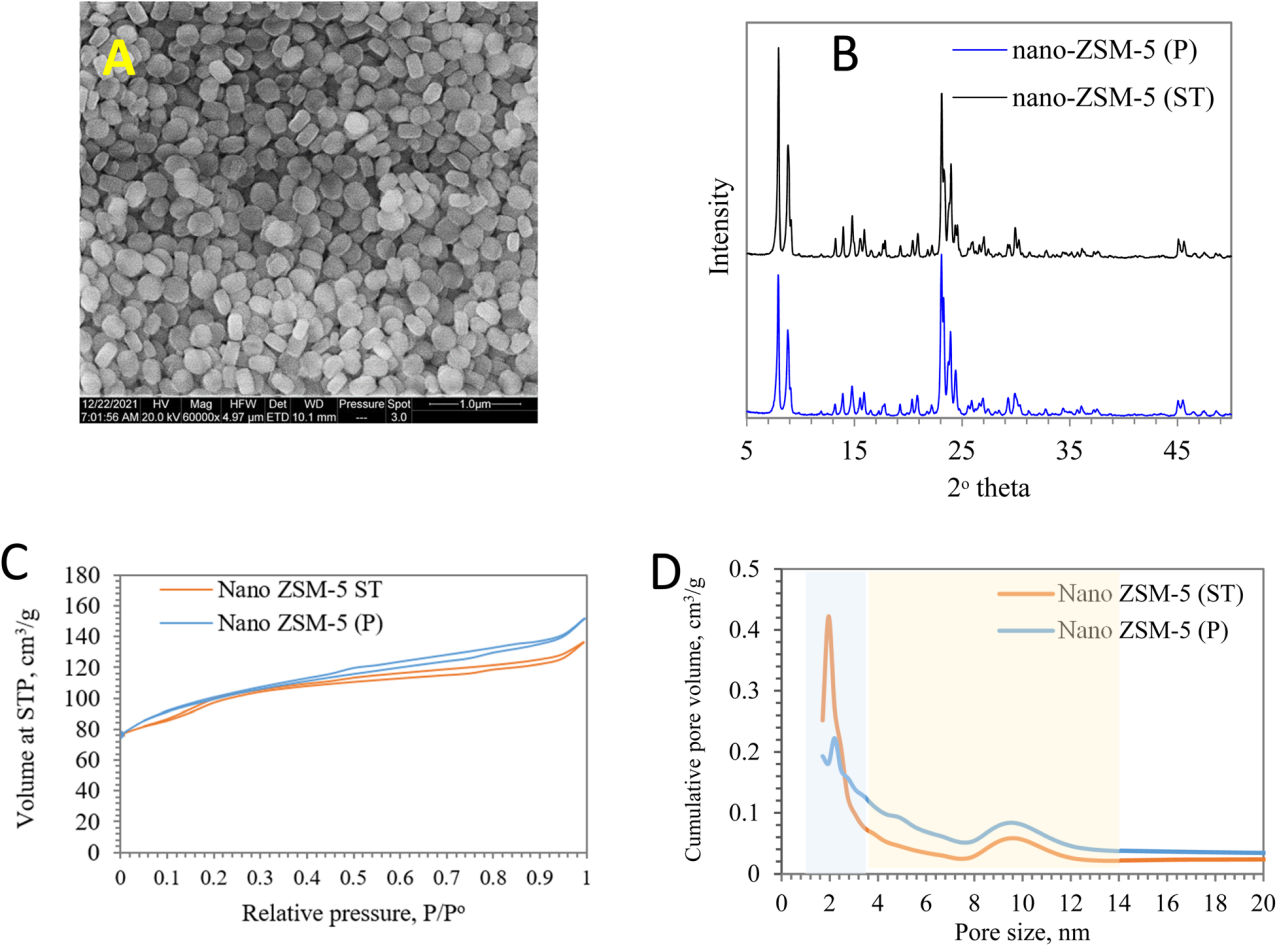
[A] SEM image and [B] XRD pattern of the synthesized ZSM-5 zeolite; [C] the nitrogen absorption isotherm plots and [D] pore size distributions of nano ZSM-5 before and after steam treatment.

The SEM topography of ZSM-5 zeolite showed semi-round, rectangular and semi-hexagonal shapes, similar to the observation by Diko *et al.*,^40^ in the range of 142–308 nm, as presented in Fig. 3A. The X-ray diffraction plot of ZSM-5 zeolite showed sharp peaks at 7.7–7.9° and 22.5–25°,^41^ which are related to the main crystalline diffractions of ZSM-5, as shown in Fig. 3B. Prins *et al.* and Wang *et al.* observed that the XRD peak intensity of dealuminated zeolite was slightly affected.^42,43^ The percentage degree of relative crystallinity was calculated according to the below equation described by Yan-feng *et al.*(7):^44,45^7
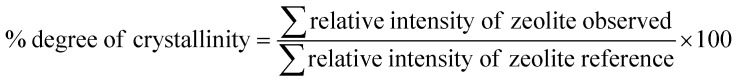


The degree of crystallinity of steam-treated nano ZSM-5 zeolite was slightly affected by the steam treatment and reduced to around 97.3% in comparison with the parent sample. Similarly, Yan-feng *et al.* reported that treating ZSM-5 (Si/Al = 25) with steam decreased the crystallinity to 94% due to the higher content of alumina.^44^ Meanwhile, Yamaguchi *et al.* reported that for ZSM-5 with a Si/Al ratio of 60, the relative crystallinity did not change after steam cracking for 16 hours due to low alumina content.^20^

**Fig. 4:**
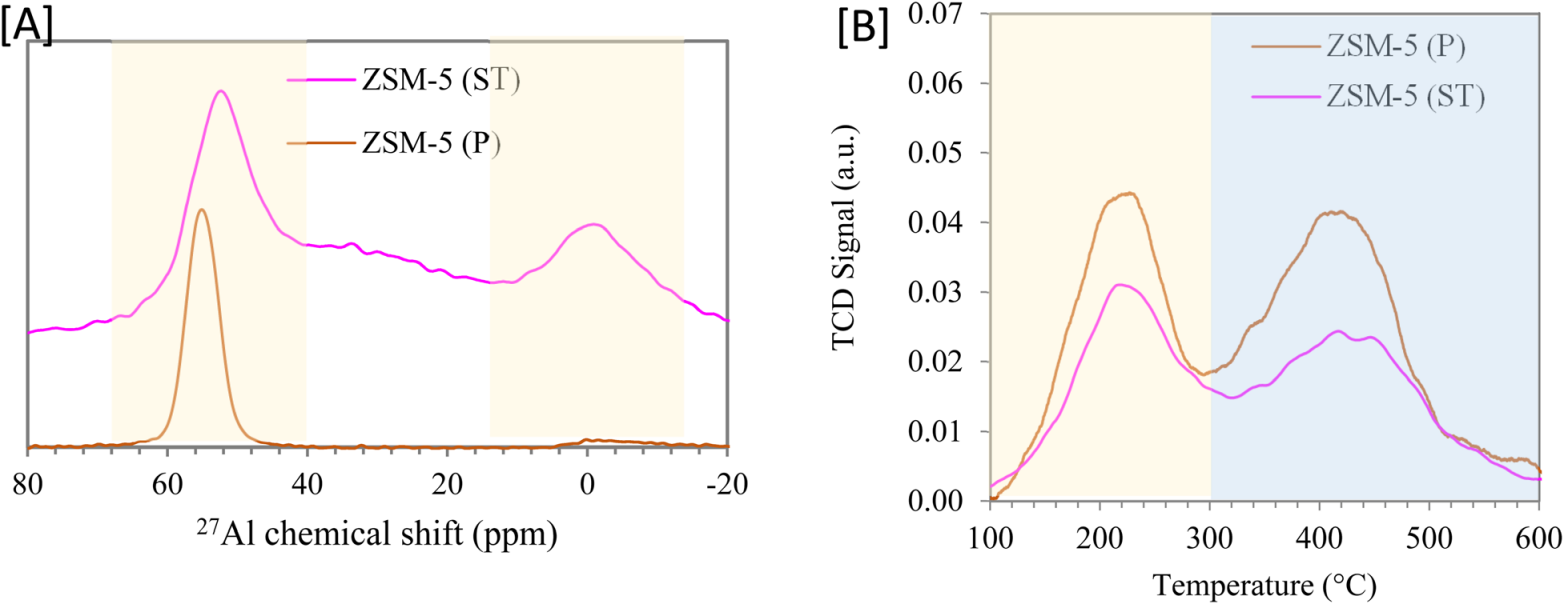
[A] The ^27^Al-NMR analysis and [B] NH_3_-TPD analysis of nanoscale ZSM-5.

Furthermore, the nitrogen adsorption analysis of the synthesized ZSM-5(P) zeolite was carried out, and the obtained BET surface area and pore volume were in the range of 376.1 m^2^ g^−1^ and 0.304 cm^3^ g^−1^, respectively (Table 1). However, after steam treatment, the textural properties of ZSM-5 were altered,^43^ and the surface area was slightly reduced to 333.1 m^2^ g^−1^. On the other hand, the pore volume of ZSM-5(ST) increased to 0.359 cm^3^ g^−1^, and this is attributed to the dealumination resulting from steam treatment. As a result of dealumination, the extra framework aluminium was observed in the Al-NMR analysis (Fig. 4A). The nitrogen adsorption–desorption isotherm was a combination of type I and type IV isotherms (Fig. 3C), and parent and steam-treated ZSM-5 samples showed hysteresis loops apparently at a relative pressure of 0.5–0.9. However, the surface area of steam-treated ZSM-5 was slightly affected, as represented by the prolonged hysteresis loop between 0.1 and 0.3. Similarly, the external pore volume after steam treatment increased from 0.186 to 0.250 cm^3^ g^−1^, and the BJH (Barrett–Joyner–Halenda) method was utilized to determine the pore volume distribution, which was altered by steam treatment^46^ and derived more pores with volume between 2 and 4 nm (Fig. 3D). This considerably large extension of pores is essential to increasing the number of pore mouths in zeolite for reactant diffusion.^47^ The Al framework of the parent synthesized nanoscale ZSM-5 and the steam-treated sample are illustrated in Fig. 4A. It could be observed from the ^27^Al MAS NMR analysis that the synthesized ZSM-5 consisted mainly of a tetrahedrally coordinated framework of the aluminum species (Al^IV^) (∼50–60 ppm) along with a minor peak associated with the extra-framework octahedral aluminum species (∼−10 to 40 ppm).^48^ On the other hand, steam-treated ZSM-5 exhibited a substantial increase in the extra-framework octahedral aluminum species, which can contribute to a reduction in catalyst surface acidity, as reported by Pu *et al.*^49^

**Table 1:** The textural properties of the synthesized nanoscale ZSM-5 zeolites

Sample	Surface area^a^, m^2^ g^−1^	Surface area microporous^b^, m^2^ g^−1^	Surface area mesoporous, m^2^ g^−1^	Pore volume^a^, cm^3^ g^−1^	Microporous pore volume^b^, cm^3^ g^−1^	External pore volume^b^, cm^3^ g^−1^
Nano ZSM-5(P)	376.1	223.8	152.3	0.304	0.118	0.186
Nano ZSM-5(ST)	333.1	207.6	125.5	0.359	0.109	0.250

a The BET (Brunauer, Emmett and Teller) method.

b The *t*-plot method.

### 3.2 The surface acidity of ZSM-5

The surface acidity of the parent and steam-treated ZSM-5 zeolites was measured by NH_3_-TPD and Py-FTIR, as shown in Fig. 4B and Table 2. The total surface acidity of the parent was found to be around 0.70 mmol g^−1^, which declined by 30% to 0.49 mmol g^−1^ after steam treatment. In addition, the measure of strong acid sites obtained at high temperatures (325–600 °C) reduced by 40% after steaming from 0.38 to 0.23 mmol g^−1^ as a result of the alteration of framework alumina to extra-framework alumina.^49^ The NH_3_-TPD method provides data on the total acidity and measure of strong and weak acid sites but doesn't provide information on the type of acid sites. In parallel, Fourier transform infrared (FTIR) with pyridine adsorption and desorption at 150 °C (ref. 50 and 51) was utilized to measure the concentration of Brønsted and Lewis acid sites, as shown in Table 2. The effect of catalyst steam treatment was clearly evident from the reduction in the availability of Brønsted acidic sites associated with the presence of the extra-framework aluminum in the framework, reflected in the overall decline in the Brønsted/Lewis acidic site ratio from 3.8 to 2.5.

**Table 2:** The surface acidity of nanoscale ZSM-5: NH_3_-TPD and Py-FTIR analyses

Catalyst	NH_3_-TPD desorption, mmol g^−1^	NH_3_-TPD low strength acidity^b^, mmol g^−1^	NH_3_-TPD high strength acidity^c^, mmol g^−1^	*C*_L_ d, mmol g^−1^	*C*_B_ e, mmol g^−1^	Total acidity, mmol g^−1^	B/L^f^, ratio
ZSM-5(P)	0.70	0.32	0.38	0.051	0.195	0.246	3.8
ZSM-5(ST)^a^	0.49	0.26	0.23	0.050	0.127	0.177	2.5

a ST: treated with steam.

b Low-strength acidity temperature 60–310 °C.

c High-strength acidity temperature 325–600 °C.

d Lewis acid sites.

e Brønsted acid sites.

f Brønsted/Lewis ratio.

### 3.3 Catalytic cracking of dodecane to olefins

#### 3.3.1 Catalytic cracking in the absence and presence of steam

**Fig. 5:**
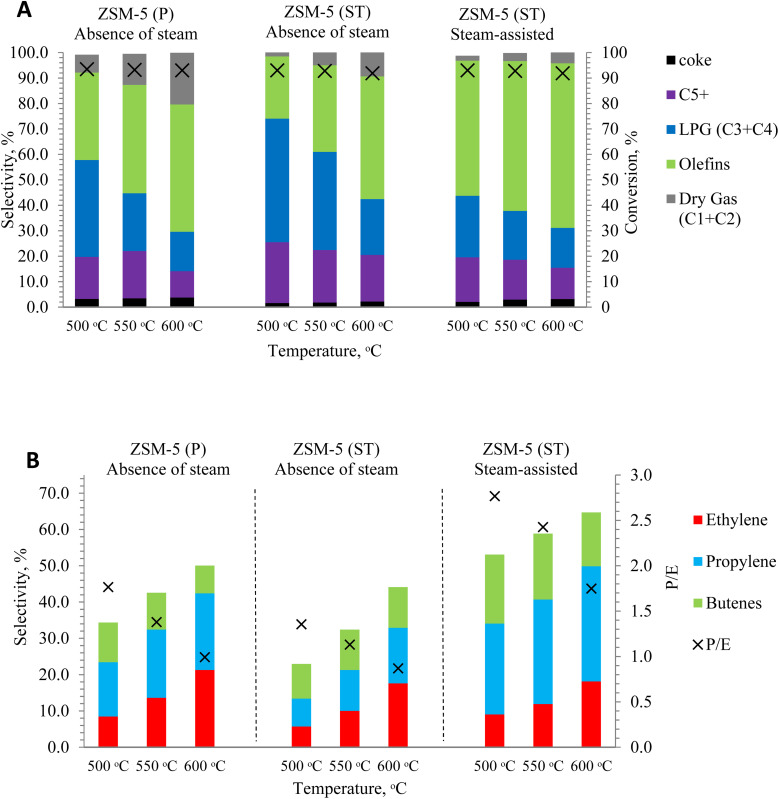
[A] Conversion and product selectivity, [B] olefin product selectivity and P/E ratio of the catalytic cracking reaction of dodecane in the absence and presence of steam at 500, 550, and 600 °C using nanoscale ZSM-5 zeolite.

The main focus of this study was to investigate the effect of steam on the catalytic cracking of dodecane toward improving light olefin production *via* the shorter diffusion path lengths of nanoscale ZSM-5. Fig. 5A shows dodecane conversion in the presence and absence of steam over nanoscale ZSM-5 at various reaction temperatures, and high conversion (>90%) was achieved in all cases. High gas selectivity (dry gas, LPG and light olefins) was obtained, which increased with the increase in the cracking temperature under both steam-assisted and steam-free conditions (Fig. 5A). As reported in earlier studies, the monomolecular reaction pathway is favored along the short path length of the nano ZSM-5 pores,^52,53^ and this effect is particularly enhanced with the pore shape selectivity of nano ZSM-5, which plays an important role in achieving higher selectivity towards light olefins.^54^ Similar observations were made by Babitz *et al.*^26^ during the catalytic cracking of the smaller hydrocarbon chain of hexane to light olefins, and they confirmed that higher temperatures strongly favor the monomolecular cracking pathway in the ZSM-5 zeolite.

In the absence of steam, catalytic cracking exhibited a noticeable increase in dry gas selectivity from 7% to 20.3% with the increase in cracking temperature from 500 °C to 600 °C. Dry gas selectivity was altered while using steam-treated ZSM-5(ST), decreasing to 3.2–11.6% as the cracking temperature increased. On the other hand, catalytic cracking in the presence of steam resulted in a substantial reduction in dry gas selectivity, resulting in values in the range of 2–5.1% as the cracking temperature increased from 500 °C to 600 °C. These observations are in concurrence with earlier studies on the catalytic cracking of heptane (*n*C_7_) to light olefins *via* ZSM-5 by Corma *et al.*^11^ Furthermore, the small crystal size along with the short path length of nanoscale ZSM-5 contributed to improving the deactivation rate and limiting coke generation.^11,55^ Specifically, it was observed that selectivity towards coke in the absence of steam was in the range of 3.2–3.8%, while the coke selectivity obtained with steam-treated ZSM-5(ST) in the absence and presence of steam was reduced slightly to 2–3.2% as the cracking temperature increased from 500 °C to 600 °C, respectively.^56,57^ In terms of the production of light olefins, the steam-assisted reaction resulted in high olefin selectivity due to more β-scission cracking and favored the monomolecular reaction pathway, because of which the olefin selectivity increased from 53.1% to 64.7% with an increase in the temperature from 500 to 600 °C, respectively. These values are higher than that achieved during dodecane conversion in the absence of steam by ZSM-5(P) and ZSM-5(ST), whose olefin selectivity values were 34.3–50% and 24.4–48.2%, respectively.

Accordingly, it is evident that the steam-assisted process has a favorable effect on increasing the gas yield, including an overall increase in olefin selectivity, during the catalytic cracking of dodecane over nanoscale ZSM-5 zeolite. However, higher ethylene selectivity was achieved in the absence of steam and the value increased from 8.5% to 21.3% upon increasing the cracking temperature from 500 to 600 °C, respectively. The increase in ethylene selectivity was accompanied by an increase in dry gas selectivity, which increased from 7% to 20.3% upon increasing the temperature from 500 to 600 °C, respectively, as shown in Fig. 5A and B. Therefore, the obtained propylene/ethylene ratio (P/E) over nanoscale ZSM-5(P) indicated that catalytic cracking towards olefins proceeded differently in the presence of steam *via* ZSM-5(ST). It was found that in the absence of steam, a P/E ratio of 1.8 was obtained, which decreased with increasing reaction temperature, suggesting that more ethylene was produced at higher temperatures. In contrast, the presence of steam in the reaction resulted in higher propylene selectivity and a high P/E ratio of around 2.8 at 500 °C, which decreased with increasing temperature and reached ∼1.7 at 600 °C. Overall, steam-assisted catalytic cracking *via* the pores of nano ZSM-5(ST) with a short diffusion path length delivered higher selectivity towards propylene and butenes (1-butene, *cis*-2-butenes, *trans*-2-butenes, isobutene) than catalytic cracking in the absence of steam. Although the acidity of nano-ZSM-5 and the associated properties promoted the dodecane conversion levels both in the presence and absence of steam, the concurrent flow of steam in the reaction affected the pore shape selectivity of the catalyst and altered the mechanism more toward the β-scission reaction^59^ and achieved higher selectivity towards olefins.^58^ However, in the absence of steam, the carbenium ions in ZSM-5(P) promoted higher C–C cracking through the β-scission mechanism and made more dry gas and ethylene products, especially at higher temperatures. On the other hand, dodecane cracking *via* ZSM-5(ST) in the absence of steam achieved low olefin selectivity and an increase in LPG products (propane and *n*-butane, and iso-butane), indicating that the zeolite pores inclined toward the hydrogenation reaction route of olefin to LPG.

#### 3.3.2 The lump kinetics of catalytic cracking

**Fig. 6:**
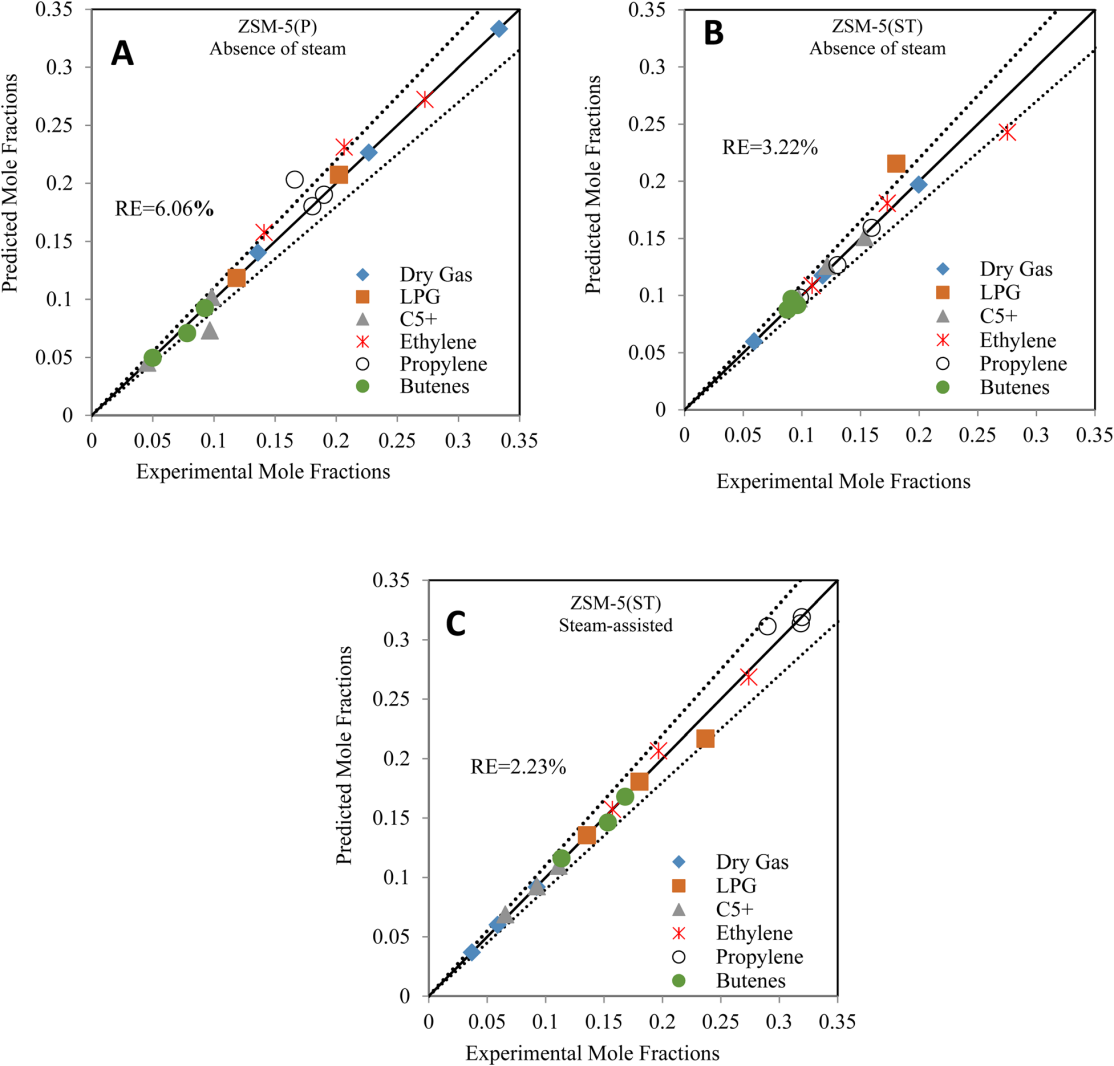
[A] Comparison between the experimental and predicted products of *n*-dodecane cracking using ZSM-5(P) in the absence of steam. [B] Comparison between the experimental and predicted products of *n*-dodecane cracking using ZSM-5(ST) in the absence of steam. [C] Comparison between the experimental and predicted products of steam-assisted *n*-dodecane cracking using ZSM-5(ST).

The kinetic analysis was performed based on the seven-lump model to study the various reaction pathways and the effect of shape selectivity inside the short diffusion path lengths of the nano ZSM-5(P) and ZSM-5(ST) pores during the catalytic cracking of dodecane in the absence and presence of steam. Higher selectivity of ethylene and dry gas (methane and ethane) was achieved in the absence of steam with ZSM-5(P), while the altered pores of steam-treated ZSM-5(ST) achieved low dry gas and favoured the hydrogenation reaction of propylene and butenes toward LPG product formation. In contrast, the catalytic cracking and reactions inside the pores proceeded differently in the presence of steam, resulting in higher propylene and butene yields with lower selectivity towards dry gas. Accordingly, the seven-lump kinetic model and the associated twenty-seven rate constants were calculated using the experimental data obtained from the catalytic cracking of dodecane. The estimated values of pre-exponential factors and activation energies in the absence and presence of steam are presented in Fig. 6A–C. The experimental and calculated product yield mole fractions were found to be within the same range, with the relative errors (eqn (6)) estimated around 6.06%, 3.22%, and 2.23% in the absence of steam while using ZSM-5(P) and ZSM-5(ST) and in the steam-assisted process using ZSM-5(ST), respectively.

**Fig. 7:**
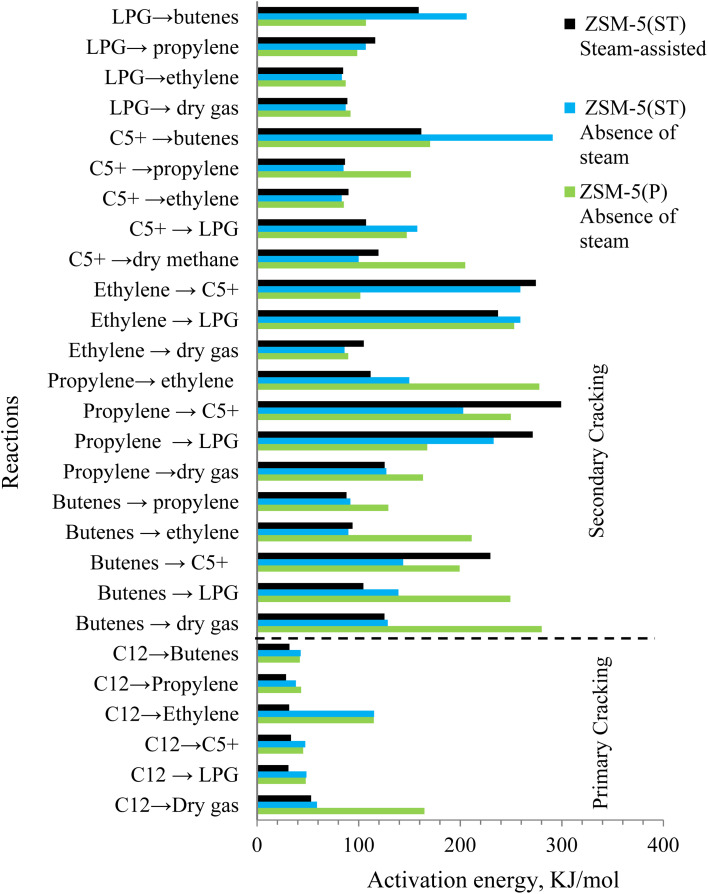
The activation energy of the reaction routes of primary and secondary cracking of dodecane in the absence of steam using ZSM-5(P) and ZSM-5(ST) and the steam-assisted conversion of dodecane using ZSM-5(ST).

**Fig. 8:**
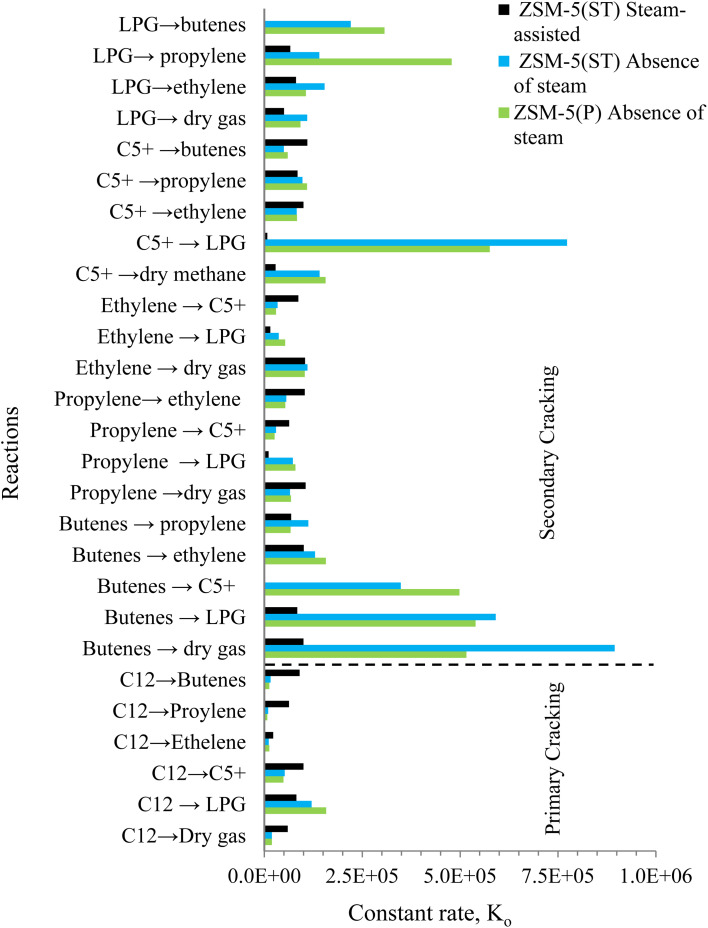
Reaction rate constants of the reaction routes of primary and secondary cracking of dodecane in the absence of steam using ZSM-5(P) and ZSM-5(ST) and the steam-assisted conversion of dodecane using ZSM-5(ST).

Accordingly, the activation energies and rate constants of the 27 reaction routes *via* the zeolite pores of nanoscale ZSM-5(P) and ZSM-5(ST) are presented in Fig. 7 and 8, respectively. Noticeably, the values of the activation energies of the reactions over ZSM-5(P) in the absence of steam were a little higher than those in the steam-assisted and steam-free conditions over ZSM-5(ST) for the primary catalytic cracking of dodecane-to-C5+, LPG, ethylene and dry gas, as shown in Fig. 7. This indicates that pore shape selectivity causes a slightly different C–C bond breaking of dodecane *via* carbenium ions through the β-scission mechanism.^59^

Similar observations were made by Corma *et al.*,^11^ who reported that the presence of steam contributes to lowering the activation energy of heptane conversion. In comparison, with dodecane (*n*C_12_), which is a longer chain molecule than heptane (*n*C_7_), the reactions occurring inside the pores apparently lead to a greater variety of byproducts compared to the fewer byproducts produced from *n*-heptane conversion. The activation energies of the secondary catalytic cracking pathways demonstrated different behavior and were higher than those of the primary catalytic cracking pathways. Variation in activation energy was observed at least for twenty-one reaction routes of the intermediate byproducts in the presence of steam *via* the pores of nanoscale zeolite with shorter diffusion lengths, which would promote selectivity toward different products than those obtained from catalytic cracking in the absence of steam. The secondary cracking reaction routes proceed *via* the β-scission mechanism, which leads to changes in both the activation energy and reaction rate of the intermediates, leading to increased production of lighter fractions, mainly in the form of dry gas, olefins and LPG (Fig. 8).

In the absence of steam *via* ZSM-5(P) and ZSM-5(ST), the rate constants for LPG and C5+ formation were simultaneously higher *via* the secondary cracking route and indicated active hydrogen transfer reactions *via* the zeolite pores^60^ unlike the process in the presence of steam. The rate constants for dry gas formation were simultaneously higher during primary and secondary cracking *via* ZSM-5(P) than those in the presence of steam. In contrast, ZSM-5(ST) derived low rate constants for dry gas in the absence and presence of steam while proceeding through the C_12_-to-dry gas, C5+-to-dry gas, butenes-to-dry gas and propylene-to-dry gas routes, as illustrated in Fig. 8. In the absence of steam, the kinetic analysis of ZSM-5(P) indicated that β-scission in the C5+-to-ethylene, LPG-to-ethylene, and butenes-to-ethylene routes proceeded with higher reaction rate constants compared with other routes and led to higher ethylene selectivity as the temperature of conversion was increased, which is similar to the observation from *n*-heptane steam catalytic cracking.^11^

An increase in the overall selectivity of ZSM-5(ST) towards propylene and butene production in the presence of steam was evident from the higher reaction rate constants *via* the primary and secondary cracking routes, which were higher than those of the same products obtained in the absence of steam. Through β-scission *via* the short-diffusion-length pores of nanoscale zeolite, the carbenium ions formed the essential primary cracking pathway toward promoting higher olefin production. Typically, in the presence of steam, the monomolecular reaction is favoured, hindering the hydrogenation and alkylation of olefins^58^ and lowering the formation of LPG and C5+ byproducts due to the low activation energies.^11^

In summary, steam-treated nanoscale ZSM-5 with the extra-framework alumina species maintained high activity despite the decline in surface acidity. Furthermore, the steam-assisted catalytic cracking of dodecane benefited from the short diffusion path lengths of the zeolite pores. Lower activation energies were obtained through the secondary cracking routes and higher reaction rate constants promoted higher selectivity toward light olefin products. In contrast, the hydrogenation and alkylation reactions to form LPG and C5+ or their further cracking to dry gas formation were less favored in the presence of steam.

## 4. Conclusions

The steam-treated nanoscale zeolite showed extra-framework octahedral Al in the tetrahedral zeolite framework, contributing to a moderate reduction in catalyst surface acidity mainly in the form of reduced Brønsted acid sites and eventually decreasing the Brønsted/Lewis acidity of zeolite. The short pores of nanoscale ZSM-5 demonstrated reactive catalytic cracking activity both in the presence and absence of steam, achieving similar dodecane conversion. In contrast, in the presence of steam, the reactions inside the pores proceeded differently and shape selectivity resulted in higher propylene and butenes yields and lower selectivity towards dry gas. At the same time, slight differences in the activation energies of the primary catalytic cracking reactions were observed between the processes in the presence and absence of steam, indicating that pore shape selectivity led to a slightly different C–C bond cracking route of dodecane *via* carbenium ions through the β-scission mechanism. On the other hand, lower activation energies for the secondary cracking reactions of intermediates were observed in the steam-assisted catalytic cracking process, apparently providing an essential hindrance to the hydrogenation and alkylation reactions. However, in the absence of steam, the pore shape selectivity promotes hydrogen transfer reactions with enhanced β-scission reactions and contributes to more dry gas and ethylene production. Overall, the short diffusion path length of the pores in nanoscale ZSM-5 affords higher selectivity for gas products in the steam-assisted catalytic cracking process, demonstrating favorable performance in cracking heavy naphtha towards achieving higher yield of olefins.

## Abbreviations

*F*_*i*_
: Molar flow rate
*i*
: Component (mol m^−3^)
*w*
: Catalyst weight
*r*_*j*_
: Reaction rate per catalyst mass
*s*_*i*,*j*_
: Stoichiometric coefficient
*j*
: Component participating in reaction
*R*
: Gas constant
*k* ^o^_*j*_
: Pre-exponential factor
*E*_A_
: Activation energy
MRE: Mean relative error
GC-MS: Gas chromatography mass spectrometry
TCD: Thermal conductivity detector
FID: Flame ionization detector
SEM: Scanning electron microscope
NH_3_-TPD: Temperature-programmed desorption of ammonia
exp: Experiment
pred: Predicated

## Conflicts of interest

There are no conflicts to declare.

## Supplementary Material
